# Supplementary material for: Effect of inter-layer spin diffusion on skyrmion motion in magnetic multilayers
Source: Sci Rep. 2019 Jul 3;9:9592. doi: 10.1038/s41598-019-46091-1 (PMC6610116; doi:10.1038/s41598-019-46091-1)
Supplement: Supplementary file 1 — Supplementary Material [file 41598_2019_46091_MOESM1_ESM.docx]

Effect of inter-layer spin diffusion on skyrmion motion in magnetic multilayers

Supplementary Material

Serban Lepadatu

*Jeremiah Horrocks Institute for Mathematics, Physics and Astronomy, University of Central Lancashire, Preston PR1 2HE, U.K.*

Diffusive Spin Torque

The inter-layer diffusive spin torque has the form:

| $\mathbf{T}_{Diff.}=-\left[(\mathbf{u}_{\perp}.\nabla)\mathbf{M}-\frac{\beta_{\perp}}{M_{S}}\mathbf{M}\times(\mathbf{u}_{\perp}.\nabla)\mathbf{M}\right]$ | **(**S1**)** |
| --- | --- |

This was verified in the main text by comparison of full spin transport solver simulations with simpler simulations based on the LLG equation augmented by the torque in Equation **(**S1**)**. To see directly how this torque arises we consider the drift-diffusion model for a simple N/F bilayer without the spin-Hall effect. For definitions of parameters see the main text. Within this model, the spin current crossing the N/F interface is absorbed as given by:

| $\left.\mathbf{J}_{S}.\mathbf{n}\right\vert_{N}-\left.\mathbf{J}_{S}.\mathbf{n}\right\vert_{F}=2D_{e}\left[\mathrm{Re}\left\{G^{\uparrow\downarrow}\right\}\mathbf{m}\times\mathbf{m}\times\left(\frac{\mathbf{S}_{F}}{\sigma_{F}}-\frac{\mathbf{S}_{N}}{\sigma_{F}}\right)+\mathrm{Im}\left\{G^{\uparrow\downarrow}\right\}\mathbf{m}\times\left(\frac{\mathbf{S}_{F}}{\sigma_{F}}-\frac{\mathbf{S}_{N}}{\sigma_{F}}\right)\right]$ | **(**S2**)** |
| --- | --- |

The longitudinal component of the spin current flowing perpendicular to the interface (z direction) inside the F layer is proportional to the z-direction derivative of the spin accumulation. This is negligible due to the long spin diffusion length in Co, as shown in Figure S1 where the spin accumulation is plotted perpendicular to the interface for a Pt/Co bilayer containing a skyrmion. On the other hand the transverse spin current inside the N layer is not negligible, and arises due to the decay (diffusion) of the spin accumulation generated in the F layer. This results in an interfacial spin torque given by:

| $\mathbf{T}_{S}=\frac{gD_{e}}{d_{F}}\left[\mathrm{Re}\left\{G^{\uparrow\downarrow}\right\}\mathbf{m}\times\mathbf{m}\times\left(\frac{\mathbf{S}_{F}}{\sigma_{F}}-\frac{\mathbf{S}_{N}}{\sigma_{F}}\right)+\mathrm{Im}\left\{G^{\uparrow\downarrow}\right\}\mathbf{m}\times\left(\frac{\mathbf{S}_{F}}{\sigma_{F}}-\frac{\mathbf{S}_{N}}{\sigma_{F}}\right)\right]$ | **(**S3**)** |
| --- | --- |

**Figure S1** – Spin accumulation for a Pt/Co bilayer without the spin-Hall effect, plotted along the z direction through the center of a skyrmion.


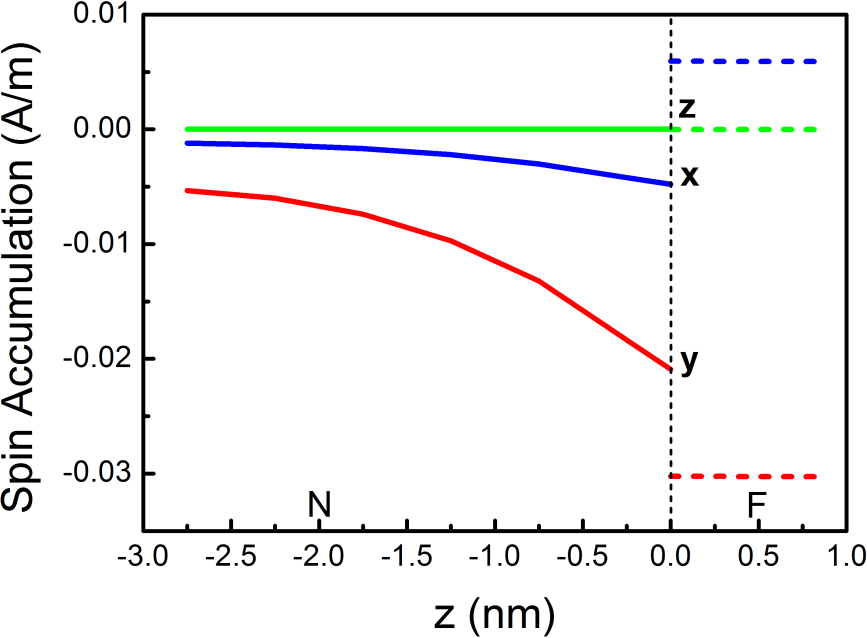


To calculate this, we need to obtain expressions for **S**_F_ and **S**_N_, the spin accumulations on either side of the interface. The spin accumulation in the F layer satisfies the following equation of motion:

| $\frac{\partial\mathbf{S}}{\partial t}=P\frac{\mu_{B}}{e}\left(\mathbf{J}_{C}.\nabla\right)\mathbf{m}-D_{e}\left(\frac{\mathbf{S}}{\lambda_{sf}^{2}}+\frac{\mathbf{S}\times\mathbf{m}}{\lambda_{J}^{2}}+\frac{\mathbf{m}\times\left(\mathbf{S}\times\mathbf{m}\right)}{\lambda_{\phi}^{2}}\right)+D_{e}\nabla^{2}\mathbf{S}$ | **(**S4**)** |
| --- | --- |

We can solve Equation **(**S4**)** for the steady state to obtain the spin accumulation in the F layer. To do this we first ignore in-plane spin diffusion, i.e. ∇^2^**S** ≅ 0, thus obtaining the spin accumulation on the F side as:

| $\mathbf{S}_{F}=a_{F}\left(\mathbf{u}.\nabla\right)\mathbf{M}+\frac{b_{F}}{M_{S}}\mathbf{M}\times\left(\mathbf{u}.\nabla\right)\mathbf{M}$ | **(**S5**)** |
| --- | --- |

Here *a_F_* and *b_F_* are given by:

| $\begin{matrix} \begin{matrix} a_{F}=\frac{\lambda_{sf,F}^{2}}{D_{e}}\frac{a^{2}+bc}{a^{2}+c^{2}} \\ \end{matrix} \\ b_{F}=\frac{\lambda_{sf,F}^{2}}{D_{e}}\frac{a^{3}+ab^{2}}{a^{2}+c^{2}} \end{matrix}$ | **(**S6**)** |
| --- | --- |

with

| $a=\frac{\lambda_{sf,F}^{2}}{\lambda_{J}^{2}}, b=\frac{\lambda_{sf,F}^{2}}{\lambda_{\phi}^{2}}, c=a^{2}+b+b^{2}$ | **(**S7**)** |
| --- | --- |

The spin accumulation in Equation **(**S5**)** leads to the Zhang-Li spin-transfer torque[[1]](#endnote-1),[[2]](#endnote-2) in the plane. In multi-layers this same spin accumulation results in vertical spin currents due to inter-layer diffusion and an interfacial spin torque as illustrated in Figure S1, which scales inversely with the F layer thickness. On the N side, without the spin-Hall effect the vertical spin current is simply proportional to the z-direction derivative of the spin accumulation, where again we ignore any in-plane spin accumulation gradients. With the composite media boundary condition in Equation **(**S2**)** and the requirement of zero spin current normal to the sample boundary, we obtain the spin accumulation on the N side of the interface as:

| $\mathbf{S}_{N}=a_{N}\left(\mathbf{u}.\nabla\right)\mathbf{M}+\frac{b_{N}}{M_{S}}\mathbf{M}\times\left(\mathbf{u}.\nabla\right)\mathbf{M}$ | **(**S8**)** |
| --- | --- |

Here we have in turn:

| $\begin{matrix} \begin{matrix} a_{N}=\frac{(1+d)d_{+}+dd_{-}}{2d^{2}+2d+1} \\ \end{matrix} \\ b_{N}=\frac{dd_{+}-(1+d)d_{-}}{2d^{2}+2d+1} \end{matrix}$ | **(**S9**)** |
| --- | --- |

with

|  | **(**S10**)** |
| --- | --- |

where $\tilde{G}_{x}^{\uparrow\downarrow}=2\mathrm{Re}\{G^{\uparrow\downarrow}\}/\sigma_{x} (x=N,F)$, and *d_N_* is the N layer thickness.

Using Equations **(**S5**)** and **(**S8**)** in Equation **(**S3**)**, Equation **(**S1**)** results, where we have:

| $\mathbf{u}_{\perp}=\mathbf{J}_{C}\frac{P_{\perp}g\mu_{B}}{2eM_{S}},$ | **(**S11**)** |
| --- | --- |

The effective parameters *P*_⊥_ and *β*_⊥_ are given as:

| $\begin{matrix} \begin{matrix} P_{\perp}=P(A+B) \\ \\ \beta_{\perp}=(A-B)/(A+B) \end{matrix} \\ \end{matrix}$ | **(**S12**)** |
| --- | --- |

where

| $\begin{matrix} \begin{matrix} A=\frac{2D_{e}\mathrm{Re}\{G^{\uparrow\downarrow}\}}{d_{F}}\left(\frac{a_{F}}{\sigma_{F}}-\frac{a_{N}}{\sigma_{N}}\right) \\ \\ B=\frac{2D_{e}\mathrm{Re}\{G^{\uparrow\downarrow}\}}{d_{F}}\left(\frac{b_{F}}{\sigma_{F}}-\frac{b_{N}}{\sigma_{N}}\right) \end{matrix} \\ \end{matrix}$ | **(**S13**)** |
| --- | --- |

The above derivation shows how the diffusive spin torque in Equation **(**S1**)** arises for an N/F bilayer under the simplifying assumption of negligible in-plane diffusion. For more complicated multilayered structures, corrections to the *P*_⊥_ and *β*_⊥_ parameters are required, however the form of the diffusive spin torque remains the same. With in-plane spin diffusion taken into account a modified in-plane spin accumulation results in the F layer[[3]](#endnote-3). The self-consistent spin transport solver used in this work takes into account all these effects, including in-plane spin diffusion in the N layers, combining both the spin-orbit torque (SOT) and diffusive spin torque into a single interfacial spin torque included in simulations. The disadvantage of this approach is increased simulation time due to the additional iterations required to solve the Poisson-type equations for the drift-diffusion model. This can increase the computation time by 5 times or more, thus simpler simulations using just the LLG equation augmented with the SOT and the diffusive spin torque in Equation **(**S1**)** are attractive.

Note, the current version of the micromagnetics software used for this work[[4]](#endnote-4) employs the successive over-relaxation algorithm with black-red ordering for parallelization to solve Poisson equations. Whilst this method is robust and able to handle arbitrary multi-layered structures and shapes, it does suffer from slow convergence for lower target solver errors. It is expected a more efficient algorithm, including the alternating direction iterative method with parallelized Thomas algorithm, a FFT-based Poisson solver, or a bi-conjugate gradient method, will significantly improve computation time in a future version.

**Figure S2** – Diffusive interfacial spin torque for a Pt/Co/Ta stack with skyrmions of different diameters as indicated, obtained using the spin transport solver and compared with Equation **(**S1**)** for P_⊥_ = 0.87 and β_⊥_ = -0.132.

**
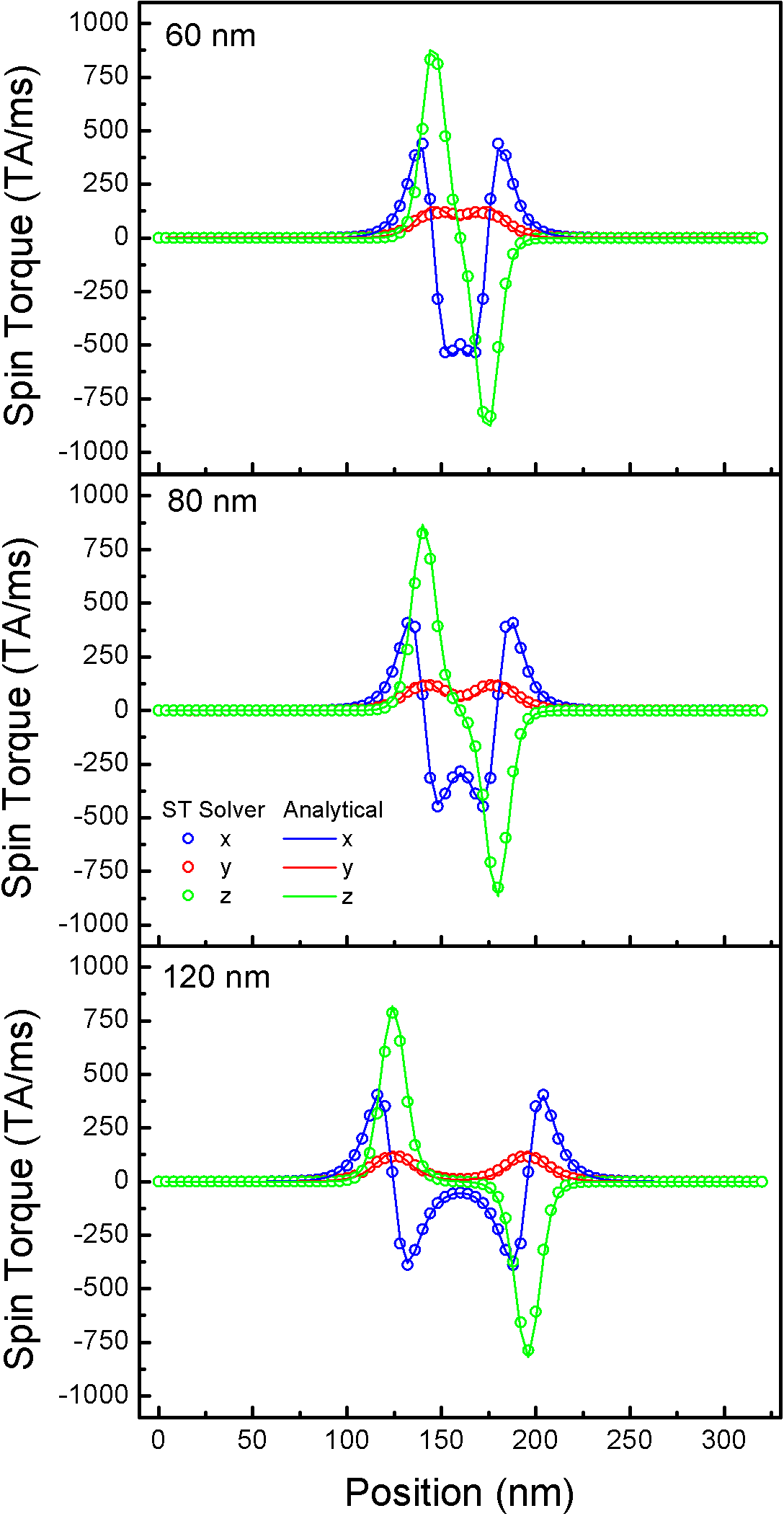
**

To use Equation **(**S1**)** in simulations without the spin-transport solver, the *P*_⊥_ and *β*_⊥_ parameters should be obtained for the multi-layered structure under study. The *P*_⊥_ and *β*_⊥_ parameters are effective parameters for the entire stack and may be obtained readily by fitting to computed diffusive spin torques with Equation **(**S1**)**. This is shown in Figure S2 for 3 different skyrmion diameters.

Current Densities in Multilayers

It was shown the effective perpendicular spin polarisation decreases with the number of repetitions, thus it is important to understand the origin of this effect. Here we take the case of 6 Pt/Co/Ta repetitions. Figure S3(a) shows the computed charge current density for this stack, where the bottom Pt layer is used to apply a voltage to the structure. Figure S3(b) shows the x component of the charge current density, plotted through the center of the stack along the vertical direction. The current densities scale with the electrical conductivity in each of the Pt, Co, and Ta layers respectively as expected. A slight decrease in current density is observed with layer number. This occurs since the voltage is applied to the stack through the bottom Pt layer only, resulting in a slight divergence of the current density with thickness. As explained in the main text this method of applying the voltage ensures that, apart from the edges of the disks, the current density is approximately uniform in the region where skyrmion motion is simulated. Figure S4 shows the resulting spin accumulation, and z-direction spin current density, also plotted through the center of the stack along the vertical direction. Here the spin-Hall effect was disabled in order to study the interfacial diffusive spin torque alone.

The decrease in effective perpendicular spin polarisation with the number of repetitions can be understood as follows. With one Pt\Co\Ta repetition, the vertical spin currents must reach zero at the outer boundaries of the Pt and Ta layers respectively. With two repetitions, the same is true for the outer Pt and Ta layers, but the situation for the inner vertical spin currents is different. The vertical spin current from the first Co layer diffuses through the inner Ta and Pt layers, reaching the second Co layer, and vice-versa. This results in a lower drop of transverse spin components for the inner interfaces, thus we obtain a relatively sharp drop in effective perpendicular spin polarisation when going from 1 to 2 repetitions, as shown in the inset of Figure 4 in the main text. As more repetitions are added, since the inner interfaces experience a lower diffusive spin torque for the reason explained above, we obtain a gradual decrease in the average diffusive spin torque, as shown in the inset of Figure 4 in the main text. This is illustrated in Figure S4, where the drop in transverse spin components for the inner interfaces is lower compared to the outer interfaces. A slight complication arises since, as seen in Figure S3, the charge current density also decreases with the layer number, thus all spin torques are gradually reduced as we go up the vertical direction. In particular the spin torques are strongest for the first Co layer.

**Figure S3** – Charge current density in a [Pt/Co/Ta]_6_ stack, showing (a) computed current density distribution where a voltage is applied to the bottom extended Pt layer, and (b) x component of current density profile plotted through the center of the stack along the z direction. The current density arrow direction is indicated by the color wheel. The horizontal dashed lines are used as guidelines.


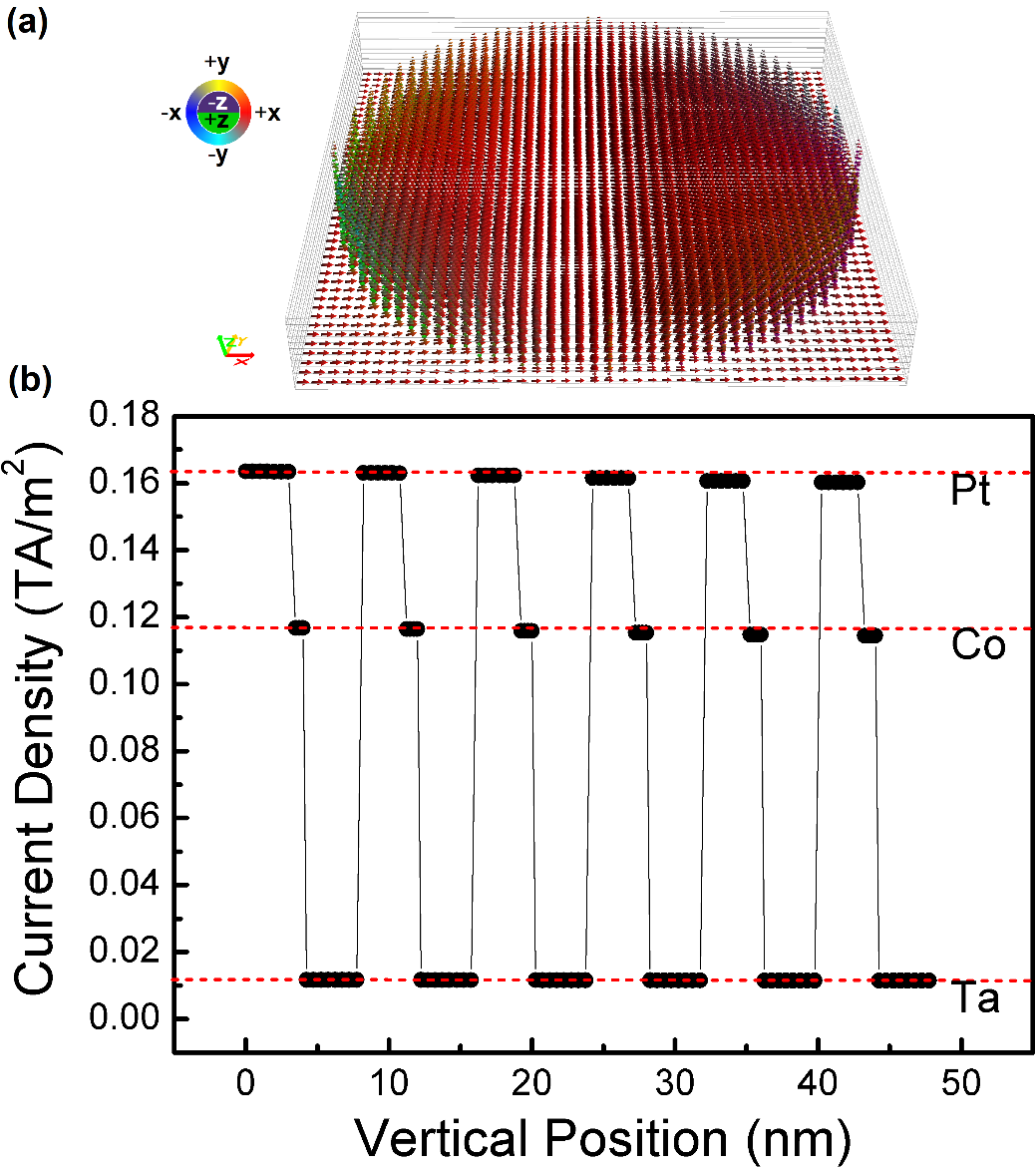


**Figure S4** – Spin transport in a [Pt/Co/Ta]_6_ stack without the spin-Hall effect, for the same current density shown in Figure S3, showing (a) x and y components of spin accumulation, and (b) x and y components of vertical spin current density. The vertical dashed lines are used as guidelines, indicating the individual layers.


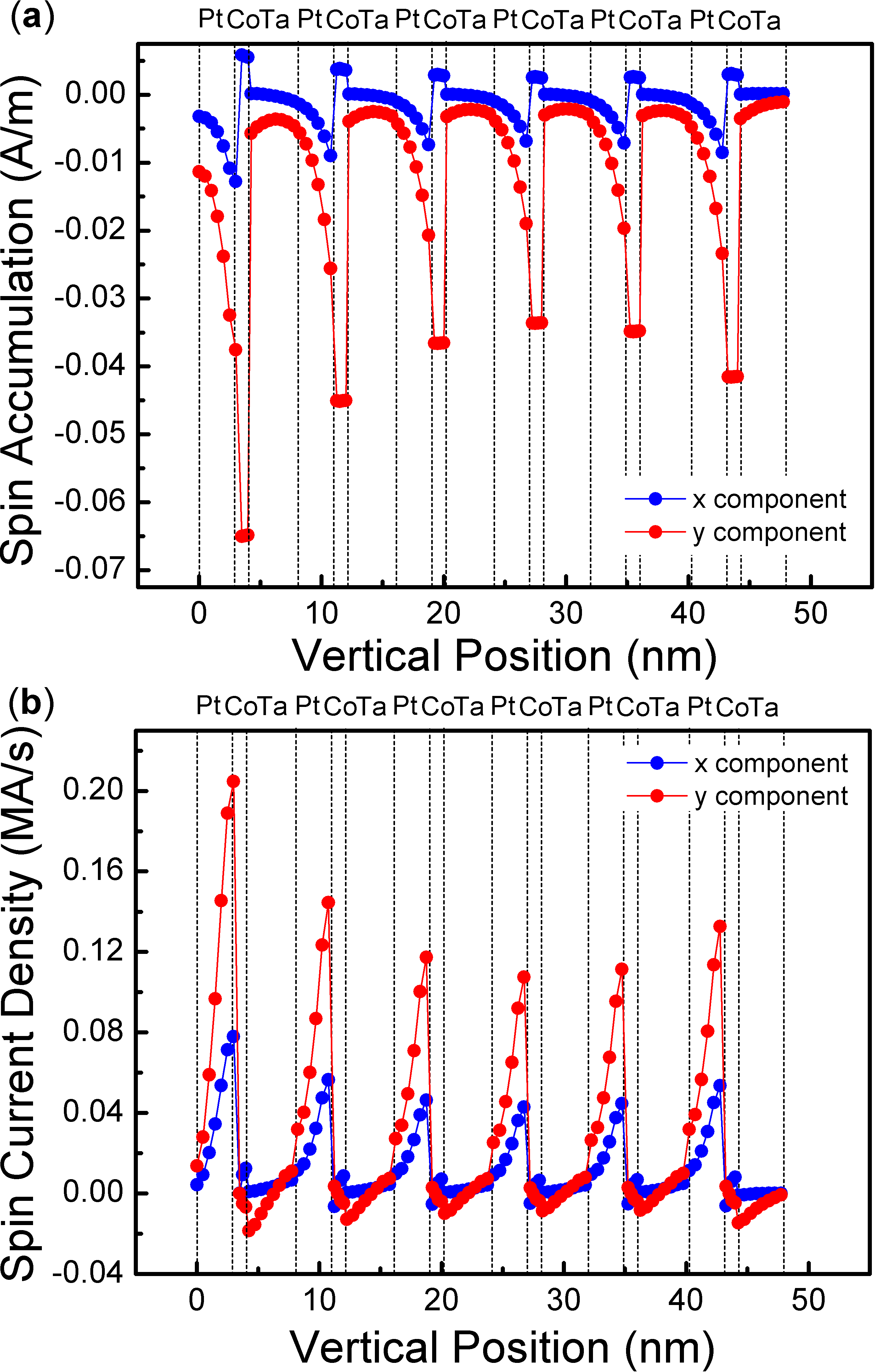


Skyrmion Structure in Multilayers

The skyrmion structure in the Pt/Co/Ta stack is verified here, taking the extreme case of 15 repetitions. The results are shown in Figure S5.

**Figure S5** – Skyrmion structure in a [Pt/Co/Ta]_15_ stack, showing skyrmions in (a) bottom layer 1, (b) middle layer 8, and (c) top layer 15. The magnetisation arrow direction is indicated by the color wheel. (d) Skyrmion profiles plotted through the center of each layer along the x axis, shown for odd-numbered layers only, plotting the z and x components of magnetisation.

**
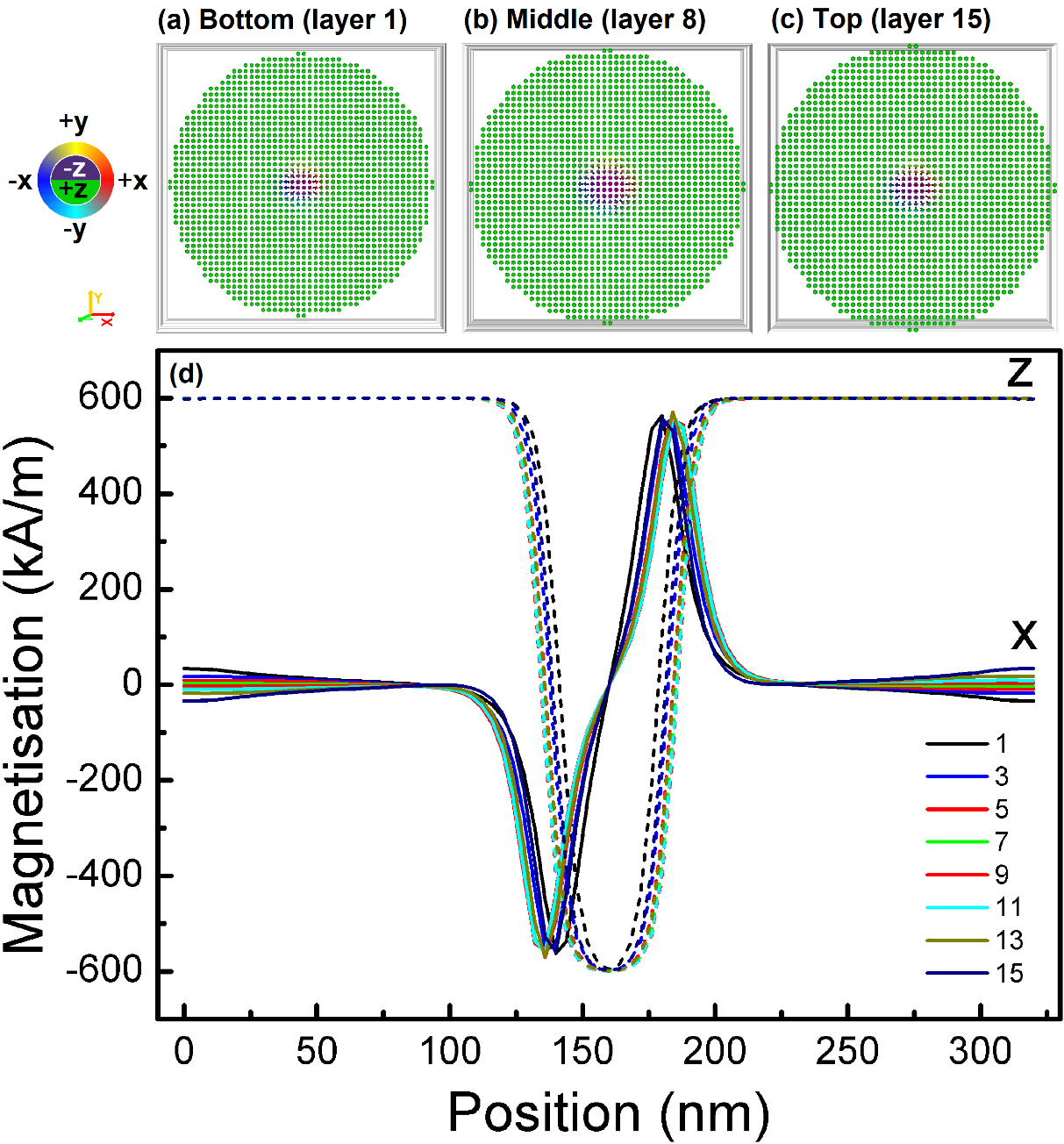
**

As can be seen in Figure S5, the skyrmion retains the same chirality throughout, with a slight change in diameter observed. In particular the diameter is largest for the middle layer and gradually decreases towards the bottom and top layers respectively. For a 60 nm average diameter skyrmion this enlargement is ~16 nm.

Finally, it was noted the skyrmion Hall angle decreases with the number of repetitions when driving skyrmions with SOT. Here we calculate the skyrmion Hall angle for the extreme case of [Pt/Co/Ta]_15_. The results are shown in Figure S6, where a 10^11^ A/m^2^ uniform current density was used. This simulation does not use the full spin transport solver, but instead the LLG equation was used, complemented by the analytical form of the SOT. The skyrmion Hall angle obtained, for magnetisation damping of 0.03, is ~76°. Even at the extreme magnetisation damping value of 0.3 the skyrmion Hall angle is still ~50°. As discussed in the main text, the combination of the diffusive spin torque and the spin-orbit torque helps to explain the observation of small skyrmion Hall angles even with moderate magnetisation damping values.

**Figure S6** – Skyrmion movement in a [Pt/Co/Ta]_15_ stack under SOT only compared to a single stack repetition, for different magnetisation damping values.


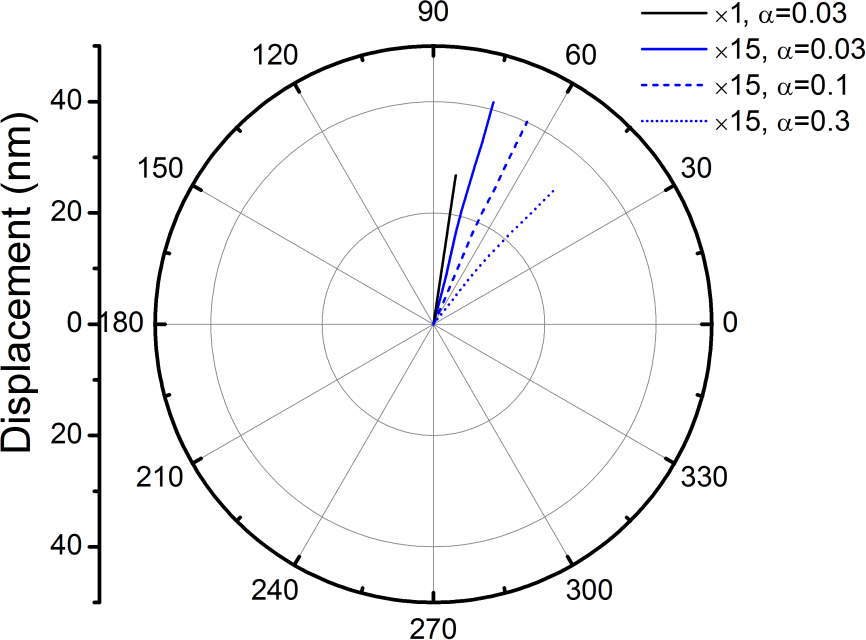


Supplementary Material References

1. . Zhang, S. & Li, Z. Roles of Nonequilibrium Conduction Electrons on the Magnetization Dynamics of Ferromagnets. *Phys. Rev. Lett.* **93**, 127204 (2004). [↑](#endnote-ref-1)
2. . Zhang, S. Levy, P.M. & Fert, A. Mechanisms of spin-polarized current-driven magnetization switching. *Phys. Rev. Lett.* **88**, 236601 (2002). [↑](#endnote-ref-2)
3. . Elías, R.G., Vidal-Silva, N. & Manchon, A. Steady motion of skyrmions and domains walls under diffusive spin torques. *Phys. Rev. B* **95**, 104406 (2017). [↑](#endnote-ref-3)
4. . Boris Computational Spintronics freely available: <https://boris-spintronics.uk/download> (21st March 2019). User Manual: DOI: 10.13140/RG.2.2.31496.88322. [↑](#endnote-ref-4)
